# Adjuvant Chemotherapy Following Complete Resection of Soft Tissue Sarcoma in Adults: A Clinical Practice Guideline

**DOI:** 10.1080/13577140220127512

**Published:** 2002-03

**Authors:** Alvaro Figueredo, Vivien H. C. Bramwell, Robert Bell, Aileen M. Davis, Manya L. Charette, The members of the cancer care Ontario practice guidelines initiative sarcoma disease site group

**Affiliations:** 1 Hamilton Regional Cancer Centre 699 Concession Street Hamilton Ontario L8V 5C2 Canada; 2 Cancer Care Ontario Program in Evidence-based Care Hamilton Ontario Canada

## Abstract

*Purpose.* To review the literature and make recommendations for the use of anthracycline-based adjuvant chemotherapy in adult patients with soft tissue sarcoma (STS).

*Patients.* The recommendations apply to patients >15 years old with completely resected STS.

*Methods.* A systematic overview of the published literature was combined with a consensus process around the interpretation of the evidence in the context of conventional practice to develop an evidence-based practice guideline.

*Results.* Four meta-analyses and 17 randomized clinical trials comparing anthracycline-based adjuvant chemotherapy versus observation were reviewed. The Sarcoma Meta-Analysis Collaboration (SMAC) was the best analysis because it assessed individual patient data and had the longest follow-up. The results of the SMAC meta-analysis together with data from more recently published randomized trials, as well as our analysis of the toxicity and compliance data, are incorporated in this systematic review.

*Discussion.* It is reasonable to consider anthracycline-based adjuvant chemotherapy in patients who have had removal of a sarcoma with features predicting a high likelihood of relapse (deep location, size >5 cm, high histological grade). Although the benefits of adjuvant chemotherapy are most apparent in patients with extremity sarcomas, patients with high-risk tumours at other sites should also be considered for such therapy.


*Sarcoma* (2002) **6**, 5–18

Taylor&Francis healthsciences

**ORIGINAL ARTICLE**

### Adjuvant chemotherapy following complete resection of soft tissue sarcoma in adults: a clinical practice guideline

ALVARO FIGUEREDO[1], VIVIEN H.C. BRAMWELL[2], ROBERT BELL[2], AILEEN M. DAVIS[2], MANYA L. CHARETTE[2], & THE MEMBERS OF THE CANCER CARE ONTARIO PRACTICE GUIDELINES INITIATIVE SARCOMA DISEASE SITE GROUP*

[1]*Hamilton Regional Cancer Centre, Hamilton, Ontario, Canada,* [2]*Cancer Care Ontario Program in Evidence-based Care, Hamilton, Ontario, Canada*

**Abstract**

*Purpose.* To review the literature and make recommendations for the use of anthracycline-based adjuvant chemotherapy in adult patients with soft tissue sarcoma (STS).

*Patients.* The recommendations apply to patients >15 years old with completely resected STS.

*Methods.* A systematic overview of the published literature was combined with a consensus process around the interpretation of the evidence in the context of conventional practice to develop an evidence-based practice guideline.

*Results.* Four meta-analyses and 17 randomized clinical trials comparing anthracycline-based adjuvant chemotherapy versus observation were reviewed. The Sarcoma Meta-Analysis Collaboration (SMAC) was the best analysis because it assessed individual patient data and had the longest follow-up. The results of the SMAC meta-analysis together with data from more recently published randomized trials, as well as our analysis of the toxicity and compliance data, are incorporated in this systematic review.

*Discussion.* It is reasonable to consider anthracycline-based adjuvant chemotherapy in patients who have had removal of a sarcoma with features predicting a high likelihood of relapse (deep location, size >5 cm, high histological grade). Although the benefits of adjuvant chemotherapy are most apparent in patients with extremity sarcomas, patients with high-risk tumours at other sites should also be considered for such therapy.

**Key words:** *soft tissue sarcoma, anthracycline, adjuvant chemotherapy, practice guideline*

### Introduction

Soft tissue sarcomas (STS) include a variety of malignant tumours affecting mesenchymal tissues of the extremities, trunk, head and neck and viscera. These tumours are rare, comprising less than 1% of all malignancies.[1] In Canada, the actual incidence in 1996 was 738 new cases.[2] Regionalized multi-disciplinary units have been recommended to provide for the best management of these patients. While wide surgical resection of tumours remains the most effective treatment, 30–50% of patients develop local recurrences and/or distant metastases, many of which eventually lead to death.[1,3,4] Predictors of disease relapse include high histological grade, size > 5 cm and deep location. These factors indicate stage III in the International Union Against Cancer (UICC) classification[5] (please see Appendix 1 for staging information). To reduce the chances of disease relapse after surgery, adjuvant radiotherapy and chemotherapy have been advocated. However, results of individual trials have been inconclusive.[1,3,4] Recently, several quantitative overviews of anthracycline-based adjuvant chemotherapy trials have been published.[6–9] The Sarcoma Disease Site Group (DSG) of the Cancer Care Ontario Practice Guidelines Initiative (CCOPGI) deemed it necessary to investigate whether anthracycline-based adjuvant chemotherapy should be recommended as part of the management of adult patients with resected STS.

*Dale Anderson, Charles Catton, Jordi Cisa, Nigel ColterJohn, Jane Curry, C. Jay Engel, Victor Fornasier, Lorraine Hands, Brian O'Sullivan, Maltibehn Patel, Shailendra Verma and Rebecca Wong also contributed to the development of this practice guideline. Please see the Cancer Care Ontario Practice Guidelines Initiative (CCOPGI) website (http:#dRwww.cancercare.on.ca/ccopgi/) for a complete list of current Sarcoma Disease Site Group members.

Correspondence to: Dr. Alvaro Figueredo, Hamilton Regional Cancer Centre, 699 Concession Street, Hamilton, Ontario, Canada L8V 5C2. Fax: +1–905–575–6326; E-mail: alvaro.figueredo@hrcc.on.ca

1357–714X print/1369–1643 online/02/010005–14 © 2002 Taylor & Francis Ltd
DOI: 10.1080/1357714022012751 2

This guideline did not consider non-anthracycline-based chemotherapy regimens or neoadjuvant chemotherapy. The guideline was developed to specifically answer the following questions:

1. What are the benefits of anthracycline-based adjuvant chemotherapy in adult patients with completely resected soft tissue sarcomas, in terms of local disease control, systemic recurrence and overall survival?
2. When these benefits are assessed in the context of expected toxicities, in what circumstances should adjuvant chemotherapy be recommended?
3. Are there any advantages in using combination versus single-agent anthracycline-based chemotherapy in the adjuvant setting?

### Methods

### *Literature search strategy*

The databases MEDLINE (Ovid) (1996–May 2001), CANCERLIT (Ovid) (1996–March 2001) and the Cochrane Library (Issue 2, 2001) were searched for trials using the terms: 'sarcoma' (Medical Subject Heading [MeSH]), 'soft tissue sarcomas' (text words), 'postoperative' (text word), 'adjuvant therapy' (text word) and 'adjuvant chemotherapy' (MeSH and text word). These terms were then combined with the search terms for the following study designs: practice guidelines, systematic reviews or meta-analyses, reviews, randomized controlled trials and controlled clinical trials. In addition, the Physician Data Query (PDQ) clinical trials database on the Internet (http://cnetdb.nci.nih.gov/trialsrch.shtml), and the proceedings of the 1997–2001 annual meetings of the American Society of Clinical Oncology (ASCO) were searched for reports of new or on-going trials. Relevant articles and abstracts were selected and reviewed by one member of the Sarcoma DSG and methodologists, and the reference lists from these sources were searched for additional trials.

### *Study selection*

Articles were selected for inclusion in this systematic review of the evidence if they met the following criteria:

1. RCTs or overviews of RCTs comparing anthracycline-based adjuvant chemotherapy to observation in patients with completely resected STS.
2. Patients were at least 15 years of age.
3. Data provided on outcomes of overall and disease-free survival.
4. Abstracts of trials were considered.

### *Synthesizing the evidence*

The intent was to perform a meta-analysis of the major outcomes, disease relapse and survival. If a previous meta-analysis had good methodology, those published results were used as evidence. To investigate outcome results not reported by previous meta-analyses (i.e., single-agent doxorubicin versus combination chemotherapy, toxicity, compliance), the data of previous meta-analyses or of individual RCTs were reanalyzed by the Sarcoma DSG using the software package Metaanalyst[0.098] (provided by J. Lau, Boston, MA, USA). To estimate overall effects, and to maintain uniformity with previous analyses, the odds ratio (OR) (95% confidence intervals [CI]) is reported according to the fixed effects model of Mantel-Haenzel and Peto. Estimates for OR > 1.0 favour the control group (observation), whereas OR < 1.0 favour the treatment group (adjuvant chemotherapy).

### *Guideline development process*

This guideline was developed by the CCOPGI Sarcoma DSG, using the methodology of the Practice Guidelines Development Cycle.[10] Evidence was selected and reviewed by one member of the Sarcoma DSG and methodologists. The guideline is a convenient and up-to-date source of the best available evidence on adjuvant chemotherapy following complete resection of STS in adults, developed through systematic reviews, evidence synthesis and input from practitioners in Ontario. It is intended to enable evidence-based practice. The Practice Guidelines Initiative is editorially independent of Cancer Care Ontario and the Ontario Ministry of Health and Long-Term Care.

Practitioner feedback was obtained through a mailed survey consisting of items asking for ratings on the quality of the draft practice guideline, and whether the draft recommendations should serve as a practice guideline. Final approval of the original guideline report was obtained from the PGCC. The CCOPGI has a formal standardized process to ensure the currency of each guideline report. This consists of periodic review and evaluation of the scientific literature, and where appropriate, integration of this literature with the original guideline information.

### Results

### *Literature search results*

Four meta-analyses[6–9] and 17 RCTs[11–31] met the inclusion criteria and were reviewed. Fourteen of the RCTs were included in the overview by the Sarcoma Meta-Analysis Collaboration (SMAC)[11–28] which also included updated individual patient data. Three RCTs[29–31] were published after the SMAC meta-analysis and are discussed in this overview. The chemotherapy regimens used in the RCTs are described in Appendix 2.

*Meta-analyses*

The first quantitative overview by Jones *et al.*[6] was literature-based and reported in abstract form only. It included 13 published trials of doxorubicin-containing adjuvant chemotherapy in resected STS at any location. Eligible trials compared adjuvant chemotherapy to observation. Statistically significant benefits favouring adjuvant chemotherapy were observed for overall survival (9% absolute benefit; $p = 0.016$), time to local recurrence ($p = 0.0003$) and metastases ($p = 0.0016$). There was also a suggestion of increased survival benefit for combination chemotherapy versus doxorubicin alone, and of decreased local recurrence when chemotherapy was combined with radiation therapy (RT). The authors found that reporting of outcomes was variable and suggested a centralized registry with standardized reporting of results for individual patient data assessment.

The second literature-based meta-analysis by Zalupski *et al.*[7] was limited to nine published trials of adjuvant chemotherapy in patients with STS of the extremities. Adjuvant chemotherapy significantly improved both overall survival and disease-free survival rates by 10 and 15%, respectively, compared to observation.

Finally, the third literature-based meta-analysis by Tierney *et al.*[8] included 15 published trials comparing adjuvant chemotherapy to no chemotherapy in resected STS of any location.[11,14,16,18,20–28] A significant improvement in overall survival (12% at 5 years; $p = 0.0002$) was noted for the treated patients. Because of concerns with potential biases in published data and the inability to investigate patient subgroups, a meta-analysis using individual patient data was proposed.

This task was undertaken by the Sarcoma Meta-analysis Collaboration[9] consisting of European and North American investigators. Although the above three previous literature-based meta-analyses[6–8] had suggested significant improvements in overall survival, disease-free survival and local recurrence with the use of adjuvant chemotherapy, the conclusions of these analyses were weakened by the possibility of publication biases and relatively short follow-up times in the published results. Therefore, the collaborative effort was to include both published and unpublished results as well as updated individual patient data, in order to obtain more reliable results and to enable subgroup analyses.

The search for data was broad; it included the MEDLINE, CANCERLIT and EMBASE databases for published material, the UK Committee on Cancer Research of Clinical Trials and the US PDQ of Clinical Protocols for unpublished trials. The search extended from 1966 to 1996. The terms used in the search were not described. Reference lists of publications were also examined. Eligible studies randomly assigned patients with localized resectable STS to receive adjuvant chemotherapy or no chemotherapy. Potential biases were avoided by including published and unpublished results and by updating individual patient data through the original investigators. The methods for combining data were clearly reported and appeared appropriate. Heterogeneity of results was investigated. The conclusions were supported by the data analysis, including clinically relevant subgroup analyses.

The overall sample included 1568 patients in 14 RCTs of doxorubicin-based adjuvant chemotherapy.[11–28] The published studies are described in Table 1. One unpublished trial by the Swiss Group for Clinical Cancer Research (SAKK) was also included in the SMAC meta-analysis. The chemotherapy regimens have been described in detail in Appendix 2. The majority of patients (74%) were 15–60 years of age, and 54% were female. Seventy-four percent of patients had primary tumours, and 11% had resected recurrent tumours. The disease affected the extremities in 58% of patients, the trunk in 12%, the uterus in 17% and other sites in 10%. The most common histological types of tumours were malignant fibrous histiocytoma (20%), leiomyosarcoma (12%), synovial sarcoma (10%) and liposarcoma (9%). The histological type was not available in 18% of cases. Most tumours (67%) were of high-grade malignancy, but in 28% of cases grade was not available. Complete tumour resections were done in 76% of patients, marginal resections were performed in 15% of the cases and assessment was not available in 9% of cases. Radiotherapy was used in 47% of cases.

The adjuvant chemotherapy consisted of doxorubicin alone in six RCTs comprising 727 patients, and of doxorubicin combined with other drugs in eight RCTs comprising 844 patients. The drugs added to doxorubicin were cyclophosphamide in seven trials, vincristine in four trials, dacarbazine in three trials, methotrexate in three trials, actinomycin D in two trials and ifosfamide in a single small unpublished trial (SAKK) (Table 1).

All reported results were given as OR, hazard ratios, or as risk differences (RD) with 95% CI using the fixed effects model of Peto. Potential heterogeneity of results was explored and no significant ($p < 0.10$) values were observed. One study[13] recorded recurrence, but did not distinguish between local and distant recurrence and, therefore, was not included in the analyses of these outcome measures[9].

In patients receiving adjuvant chemotherapy, there was a non-significant trend for increased overall survival (Fig. 1 and Table 2) with a RD of 4% (95% CI, -1 to 9%) when compared with patients who received no adjuvant chemotherapy. Disease-free survival was significantly increased in treated patients with an absolute RD of 10% (95% CI, 5–15%; $p = 0.0001$) at 10 years. This difference was mostly due to patients who were free of metastases, in whom

**Table 1.** *Randomized trials of doxorubicin-based adjuvant chemotherapy in resected soft tissue sarcomas in adults (data from 13 published studies included in SMAC meta-analysis)*

| | | Chemotherapy | | | | | No. of patients assigned to each group (entered/evaluable) | |
|---|---|---|---|---|---|---|---|---|
| Trial (Ref.) | Disease sites | Chemo. | Dose per course* | Dose intensity† | Total dose‡ | RT | Control | Dox. |
| GOG(11) | Ut | Dox | 60 | 20 | 480 | No | 112/81 | 113/75 |
| DFCI(12) | Ex,RP,HN,Tr | Dox | 90 | 30 | 450 | Yes | 22/22 | 20/20 |
| ECOG(13) | Ex,RP,HN,Tr | Dox | 70 | 23.3 | 490 | No | NA/13 | NA/17 |
| SSG(14) | Ex,HN,Br,Tr,Abd | Dox | 60 | 15 | 540 | Some | 119/88 | 121/93 |
| Rizzoli(15,16) | Ex | Dox | 75 | 25 | 450 | Some | 35/35 | 24/24 |
| IGSG(18,19) | EX,RP,HN,Tr | Dox | 70–90 | 23.3–26.7 | 450 | Some | NA/43 | NA/39 |
| MDAH(20) | Ex | VACAR | 60 | 15 | 420 | Yes | 24/23 | 22/20 |
| Mayo(21,22) | Ex,Tr | VAC/VAD | 50 | 8.3 | 200 | No | NA/31 | NA/30 |
| NCI(23,24) | Ex | AC/MTX | 50–70 | 12.5–15.6 | 550 | Yes | 28/28 | 39/37 |
| NCI(25,26) | HN,Tr,Br | AC/MTX | 50–70 | 12.5–15.6 | 550 | Yes | 14/27 | 17/30 |
| NCI(25,26) | RP | AC/MTX | 50–70 | 12.5–15.6 | 550 | Yes | 7/16 | 8/21 |
| EORTC(27) | Ex,RP,Tr,HN,Ut | CYVADIC | 50 | 12.5 | 400 | Yes | 234/172 | 234/145 |
| Bergonié(28) | Ex,RP,HN,Tr | CYVADIC | 50 | 8.33 | 500 | Yes | NA/28 | NA/31 |

Note: Abd, abdominal; AC, doxorubicin+cyclophosphamide; Br, breast; Chemo., chemotherapy; CYVADIC, cyclophosphamide+vincristine+doxorubicin+dacarbazine; Dox, doxorubicin; Ex, extremities; HN, head and neck; MTX, methotrexate+leucovorin; NA, data not available; RP, retroperitoneal; RT, radiation therapy; Tr, trunk; Ut, uterine; VAC, vincristine+cyclophosphamide+actinomycin D; VACAR, vincristine+doxorubicin+cyclophosphamide+actinomycin D; VAD, ncristine+doxorubicin+dacarbazine.
* Dose per course in mg/m$^2$.
† Dose intensity in mg/m$^2$ per week considering only the first four courses of chemotherapy.
‡ Total dose in mg/m$^2$.

there was a 10% difference (95% CI, 5–15%; $p = 0.0003$) (Table 2). The difference for local recurrence was smaller, but still statistically significant (RD, 6%; 95% CI, 1–10%; $p = 0.016$). These pooled results were not changed by excluding patients younger than 15 years of age, the presence of local recurrence or metastases, nor whether patients received induction chemotherapy.

Although tests of the overall data did not show significant statistical heterogeneity ($p < 0.10$), there was evidence of clinical heterogeneity, indicated by the wide range of age groups, tumour locations, tumour sizes, types and grades of malignancy, surgical treatments, use of adjuvant RT and regimens of chemotherapy. The results of these subgroup analyses were difficult to interpret. The SMAC authors stated 'For overall survival, there was no clear evidence to suggest that any subgroup benefited more or less from adjuvant chemotherapy ... There was some suggestion that men benefited more than women from chemotherapy [*but this was felt to be biologically implausible*]. Among patients with lesions of the extremities (376 deaths, 886 patients), the hazard ratio was 0.80 ($p = 0.029$), equivalent to a 7% absolute benefit at 10 years. This group had the clearest evidence of a treatment effect on survival. The wide confidence intervals for the other sites reflect the small numbers, and there was no clear evidence that the results differed from those for extremity sarcomas ($p = 0.58$).' There was no evidence of a differential effect of chemotherapy for any of the subgroups considered for relapse-free survival.

The conclusion of the SMAC project was that doxorubicin-based adjuvant chemotherapy, following resection of STS, is associated with a significant prolongation of the disease-free interval (mostly due to delay in metastases) and a trend for increased survival. The largest benefit in survival was in the subgroup of patients with extremity sarcomas, with an absolute 7% benefit at 10 years. Converting the RD to the number needed to treat (NNT) resulted in a value of 14 patients requiring treatment with adjuvant chemotherapy to delay one death.

### *Treatment effects according to chemotherapy used*

All of the adjuvant chemotherapy regimens investigated by the SMAC contained doxorubicin, but seven of them also included other drugs (Table 1). The dose of doxorubicin ranged from 50 to 90 mg/m$^2$ per course, with total cumulative doses between 400 and 550 mg/m$^2$. A dose–response relationship was investigated but not detected in any of the meta-analyses.[6–9] Using data from the SMAC paper,[9] a new analysis was performed evaluating the effect of treatment with doxorubicin alone versus doxorubicin combined with other drugs. For patients treated with single-agent doxorubicin versus patients who received no adjuvant chemotherapy, the mortality OR was 0.80 (95% CI, 0.60–1.07), compared with 0.89 (95% CI, 0.67–1.18) for patients on combined chemotherapy versus those randomized to observation. The OR for recurrence for patients treated with single-agent doxorubicin versus those who received

| | Events/Total entered: Chemotherapy | Events/Total entered: Control | O-E | Variance |
|---|---|---|---|---|
| **Local RFI** | | | | |
| GOG | 20/112 | 16/109 | 1.25 | 8.97 |
| MDA | 2/18 | 5/17 | -1.61 | 1.75 |
| Mayo | 4/22 | 5/23 | -0.66 | 2.24 |
| NCI4 | 2/17 | 4/8 | -2.49 | 1.11 |
| NCI5 | 8/38 | 9/41 | 0.09 | 4.22 |
| NCI6 | 0/21 | 0/20 | 0.00 | 0.00 |
| EORTC | 30/193 | 51/188 | -12.25 | 20.16 |
| DFCI/MGH | 3/21 | 3/25 | 0.23 | 1.49 |
| Bergonie | 6/28 | 8/26 | -2.31 | 3.33 |
| SSG | 19/121 | 13/119 | 2.59 | 7.94 |
| Rizzoli | 1/16 | 6/22 | -1.96 | 1.71 |
| IGSC | 6/40 | 8/46 | -0.93 | 3.49 |
| SAKK | 0/12 | 0/12 | 0.00 | 0.00 |
| *Total* | 101/659 | 128/656 | -18.05 | 56.42 |
| **Distant RFI** | | | | |
| GOG | 21/112 | 36/109 | -8.70 | 14.20 |
| MDA | 8/18 | 8/17 | -1.10 | 3.70 |
| Mayo | 6/22 | 6/23 | -0.01 | 3.00 |
| NCI4 | 4/17 | 3/8 | -1.10 | 1.38 |
| NCI5 | 12/38 | 16/41 | -1.51 | 6.98 |
| NCI6 | 7/21 | 10/20 | -2.12 | 4.20 |
| EORTC | 51/193 | 56/188 | -3.43 | 26.73 |
| DFCI/MGH | 6/21 | 7/25 | -0.20 | 3.24 |
| Bergonie | 8/28 | 14/26 | -5.00 | 5.29 |
| SSG | 41/121 | 53/119 | -7.87 | 22.97 |
| Rizzoli | 6/16 | 10/22 | -1.05 | 3.86 |
| IGSC | 4/40 | 13/46 | -4.14 | 4.24 |
| SAKK | 3/12 | 4/12 | -0.44 | 1.75 |
| *Total* | 177/659 | 236/656 | -36.65 | 101.54 |
| **Overall RFS** | | | | |
| GOG | 52/113 | 62/112 | -6.75 | 28.42 |
| MDAH | 12/18 | 15/17 | -4.65 | 5.88 |
| Mayo | 12/22 | 11/23 | 0.21 | 5.71 |
| NCI4 | 9/17 | 5/8 | -1.42 | 2.64 |
| NCI5 | 22/38 | 24/41 | -0.33 | 11.47 |
| NCI6 | 9/21 | 11/20 | -2.00 | 4.90 |
| EORTC | 92/193 | 105/188 | -13.31 | 48.84 |
| DFCI/MGH | 7/21 | 8/25 | -0.20 | 3.74 |
| ECOG | 9/24 | 11/23 | -1.70 | 4.96 |
| Bergonie | 11/28 | 19/26 | -7.60 | 6.96 |
| SSG | 65/121 | 69/119 | -5.35 | 32.72 |
| Rizzoli | 7/16 | 13/22 | -2.10 | 4.85 |
| IGSC | 14/40 | 25/46 | -5.09 | 9.73 |
| SAKK | 4/12 | 4/12 | 0.08 | 2.00 |
| *Total* | 325/684 | 382/682 | -50.21 | 172.83 |
| **Overall survival** | | | | |
| GOG | 51/113 | 55/112 | -1.37 | 26.43 |
| MDAH | 15/26 | 20/28 | -3.13 | 8.65 |
| Mayo | 14/28 | 12/29 | 1.46 | 6.45 |
| NCI4 | 9/17 | 5/8 | -1.57 | 2.54 |
| NCI5 | 22/38 | 23/41 | 1.32 | 11.15 |
| NCI6 | 8/21 | 9/20 | -1.01 | 4.21 |
| EORTC | 94/234 | 96/233 | -0.60 | 47.47 |
| DFCI/MGH | 6/21 | 7/25 | 0.15 | 3.22 |
| ECOG | 9/24 | 10/23 | -1.29 | 4.69 |
| Bergonie | 10/33 | 18/32 | -5.94 | 6.82 |
| SSG | 57/121 | 57/119 | -1.30 | 28.48 |
| Rizzoli | 12/34 | 25/43 | -5.83 | 9.19 |
| IGSC | 16/43 | 23/49 | -2.72 | 9.72 |
| SAKK | 5/14 | 3/15 | 1.55 | 1.94 |
| *Total* | 328/767 | 363/777 | -20.29 | 170.95 |

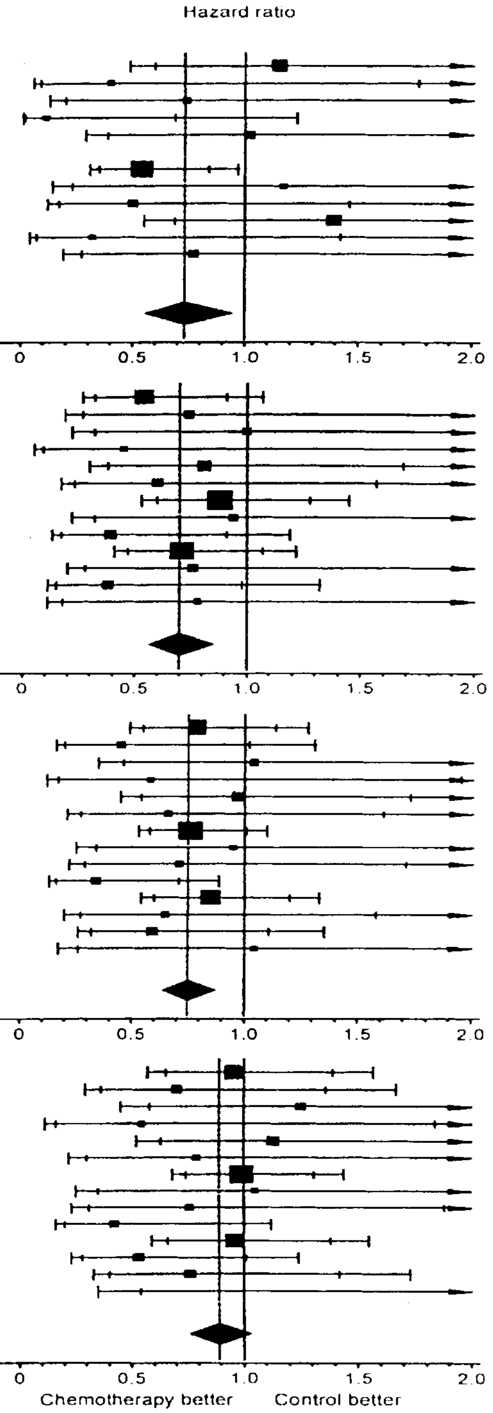

**Fig. 1.** *Meta-analysis of the effects of adjuvant chemotherapy versus observation. Squares represent hazard ratios; area is proportional to amount of information available in trial; bars represent 95% confidence interval (CI) (inner limit) and 99% CI (outer limit). Diamonds represent overall hazard ratios for results of all trials combined — extremes of diamond give 95% CI; O–E, observed–expected; RFI, recurrence-free interval; RFS, recurrence-free survival. Note: statistical heterogeneity was p>0.10 in all four analyses: local recurrence-free interval, p=0.17; distant recurrence-free interval, p=0.86; overall recurrence-free survival, p=0.75; overall survival, p=0.54. (Used with permission: Sarcoma Meta-analysis Collaboration. Adjuvant chemotherapy for localized resectable soft-tissue sarcoma of adults: meta-analysis of individual data. Lancet 1997; 350: 1647–54.)*

no adjuvant chemotherapy was 0.71 (95% CI, 0.53–0.96), similar to the OR for patients receiving doxorubicin-containing combination adjuvant therapy versus patients randomized to no adjuvant chemotherapy (OR, 0.69; 95% CI, 0.51–0.94).

*Adverse effects*

Among the 14 trials included in the SMAC, toxicity data were not available for one unpublished study (SAKK) and for one published trial.[20] Reporting of

**Table 2.** *Pooled results of adjuvant chemotherapy in adult patients with resected soft tissue sarcomas (data from the Sarcoma Meta-analysis Collaboration)*

| Survival rate | No. of trials | No. of pts. | Odds ratio (95% CI) | *P* value | Risk difference (95% CI)† |
|---|---|---|---|---|---|
| Overall | 14 | 1544 | 0.89 (0.76–1.03) | 0.12 | -0.04 (-0.09 to -0.01) |
| Overall‡ | 12 | 886 | 0.80 (NA) | 0.029 | -0.07 (NA) |
| Disease-free | 14 | 1366 | 0.75 (0.64–0.87) | 0.0001 | -0.10 (-0.15 to -0.05) |
| Without local recurrence | 13* | 1315 | 0.73 (0.56–0.94) | 0.016 | -0.06 (-0.10 to 0.01) |
| Without metastases | 13* | 1315 | 0.70 (0.57–0.85) | 0.0003 | -0.10 (-0.15 to -0.05) |

Note: CI, confidence interval; NA, data not available; pts, patients.
* ECOG trial (13) not included; data not available.
† A minus (-) sign in front of the risk difference indicates a decrease in risk for treated patients versus controls.
‡ Only patients with extremity sarcomas.

**Table 3.** *Severe toxicity of adjuvant chemotherapy in adult patients with resected soft tissue sarcomas (data from published papers)*

| Trial (Ref.) | Chemotherapy* | No. of evaluable patients | No. of toxic deaths (%) | No. of pts infected (%) | No. of pts with cardiotoxicity (%) |
|---|---|---|---|---|---|
| GOG(11) | Dox | 75 | 0 | 0 | 6 (8.0) |
| DFCI(12) | Dox | 20 | 1 (5.0) | 1(5.0) | 2 (10.0) |
| ECOG(13) | Dox | 17 | 0 | NA | 0 |
| SSG(14) | Dox | 93 | 3 (3.2) | 0 | 4 (4.3) |
| Rizzoli(15,16) | Dox | 24 | 0 | 0 | 1 (4.2) |
| ISG(18,19) | Dox | 39 | NA | 0 | 2 (5.1%) |
| All Dox alone | Dox | 268 | 4/229 (1.7) | 1/251 (0.4) | 15/268 (5.6) |
| MDAH(20) | VACAR | 20 | NA | NA | NA |
| Mayo(21,22) | VAC/VAD | 30 | 1(3.3) | 1 (3.3) | 0 |
| NCI(23,24) | AC/MTX | 37 | 0 | NA | NA |
| NCI(25,26) | AC/MTX | 30† | 1(3.3) | 2 (6.7) | 5 (16.7) |
| NCI(25,26) | AC/MTX | 21‡ | 2 (9.5) | 3 (14.3) | 3 (14.3) |
| EORTC(27) | CYVADIC | 145 | 1 (0.7) | 1 (0.7) | 2 (1.4) |
| Bergonié(28) | CYVADIC | 31 | 0 | NA | NA |
| All Dox + other | Dox + other | 314 | 5/294 (1.7) | 7/226 (3.1) | 10/226 (4.4) |
| All | Dox +/- other | 582 | 9/523 (1.7) | 8/477 (1.7) | 25/494 (5.1) |

Note: Infected, severe infections; NA, data not available.
* Chemotherapy: Please see footnote of Table 1.
† Includes 13 patients treated outside the randomized trial.
‡ Includes 13 patients treated outside the randomized trial.

adverse effects in the other trials was not standardized. Some trials reported mostly qualitative assessments of toxicity,[11–13,15,21,28] while others reported quantitative data according to standard toxicity grading scales.[14,17,23–27] Adverse effects frequently consisted of alopecia, fatigue, anorexia, nausea and vomiting, leucopenia or neutropenia, thrombocytopenia, infections, mucositis and cardiotoxicity. Alopecia was considered significant in 50%[27] to 90%[13,21,25] of patients. Nausea and vomiting was mild to moderate in some trials; it was considered severe in 22–50% of patients in other trials.[13,14,17,19] Various degrees of nadir or treatment day leucopenia or neutropenia were described in all trials, but neutropenic sepsis was uncommon and only a single patient was reported as dying due to infection.[27] Severe thrombocytopenia was rare but it contributed to death in one patient with concurrent neutropenic sepsis and multiple organ failure.[27] Cardiotoxicity was generally reported and consisted of arrhythmias and congestive heart failure, the latter leading to death in six patients.[14,19,21,25,26] While the reported adverse effects could be attributed to doxorubicin, there were other side-effects associated with other drugs. Severe peripheral neuropathy induced by vincristine was reported in four (3.2%) of 126 patients treated with cyclophosphamide, vincristine, doxorubicin and dacarbazine.[27] Cystitis was noted in four (7.8%) patients treated with doxorubicin, cyclophosphamide, methotrexate and leucovorin and concurrent RT;[25,26] another patient had reduced creatinine clearance on high-dose methotrexate.[26] Diarrhea occurred in two (8%) of 30 patients receiving vincristine, doxorubicin, cyclophosphamide and actinomycin D.[21]

The available quantitative toxicity data has been tabulated for toxic deaths and major toxic events such as severe infections and cardiotoxicity (Table 3). The overall rate of toxic deaths was nine in 523 patients (1.7%), and was the same for patients treated with doxorubicin alone or combined with other drugs. The overall rate of cardiotoxicity was 25 cases among 494 patients (5.1%). Those receiving doxorubicin alone had a 5.6% cardiotoxicity rate, while the cardiotoxicity rate for doxorubicin combined with other drugs was 4.4%. Severe infections occurred more commonly in patients receiving multiple drug chemotherapy with a rate of 3.1% (seven of 226

patients) versus 0.4% (one of 251 patients) in those receiving only doxorubicin (Table 3).

Overall, no correlation was found between chemotherapy toxicity and the use of RT (data not shown). Only three trials did not use RT[11,13,21] and another omitted the use of doxorubicin during RT.[28] Of note, however, is that one trial of combined RT and chemotherapy versus RT alone in patients with retroperitoneal sarcomas was terminated because of poor results and toxicity in the combined treatment group.[25]

### *Compliance with chemotherapy*

Compliance data were not reported in six trials,[12,13,17,20,25,26] reported in a limited manner in three trials,[15,21,28] and investigated in more detail and correlated with major outcomes in four other trials.[11,14,24,27] Compliance data were not available for the unpublished trial (SAKK) included in the SMAC meta-analysis. In Table 4, we have tabulated compliance with chemotherapy according to whether patients received any treatment, or had minor or major reductions of chemotherapy. We considered patients receiving at least four courses of chemotherapy as having a minor reduction of treatment.

Overall compliance with treatment was similar for both single-agent doxorubicin and doxorubicin combined with other drugs. Of 276 patients randomized to receive doxorubicin alone, data were not available for 76 patients. Of the data available for 200 patients, 107 (53.5%) received full dose treatment and 177 (88.5%) received at least four courses of treatment. Among 314 patients randomized to receive doxorubicin combined with other drugs, data were only available for 243 patients. Of these 243 patients, 143 (58.8%) received full treatment and 184 (75.7%) received at least four courses of chemotherapy (Table 4).

### *Recent randomized clinical trials*

A search for trials published after the SMAC overview uncovered three RCTs.[29–31] Investigators at the University Hospital in Vienna reported on 59 patients recruited into an RCT between 1992 and 1999, which was stopped because of poor accrual.[29] These patients had mostly STS of the extremities, grade II or III, and had wide or marginal resection. Post-operatively, the patients were randomized to RT alone (51 Gy hyperfractionated), or to the same RT plus dose-intensive chemotherapy with ifosfamide, dacarbazine and doxorubicin, with the support of granulocyte-colony stimulating factor (G-CSF) after wide or marginal resection.[29] After a mean observation period of 41±19.7 months, there was a non-significant decrease in local relapses, all relapses and death for the group of patients receiving adjuvant chemotherapy. In the subgroup of patients with grade III tumours, disease-free survival (but not overall survival) was significantly improved by chemotherapy ($p = 0.03$). Toxicity was moderate with no fatalities and no episodes of neutropenic sepsis or cardiomyopathy. One patient in the RT group and three patients in the RT plus chemotherapy group had local complications related to the underlying bone.

Another trial was reported by a group of Italian investigators.[30] The trial is reported for 104 patients with resected, high-grade, primary or recurrent STS of the extremities and girdles randomized to either observation or five courses of intensive adjuvant chemotherapy with epirubicin and ifosfamide supported

**Table 4.** *Compliance with adjuvant chemotherapy (data from published papers)*

| Trial (Ref.) | Chemotherapy* | Total no. of pts | No. of no treatment (%) | No. of major treatment reduction (%) | No. of minor treatment reduction (%) | No. of full treatment (%) |
|---|---|---|---|---|---|---|
| GOG(11) | Dox | 83 | 8 (9.6) | 6 (7.2) | 14 (16.9) | 55 (66.3) |
| DFCI(12) | Dox | 20 | NA | NA | NA | NA |
| ECOG(13) | Dox | 17 | NA | NA | NA | NA |
| SSG(14) | Dox | 93 | NA | 10 (10.8) | 54 (58.1) | 29 (31.2) |
| Rizzoli(15,16) | Dox | 24 | 0 | 0 | 2 (8.3) | 23 (95.8) |
| ISG(18,19) | Dox | 39 | NA | NA | NA | NA |
| All Dox-alone | Dox | 276 | 8/107 (7.5) | 16/200 (8.0) | 70/200 (35.0) | 107/200 (53.5) |
| MDAH(20) | VACAR | 20 | NA | NA | NA | NA |
| Mayo(21,22) | VAC/VAD | 30 | 0 | 5 (16.7) | 2 (6.7) | 23 (76.7) |
| NCI(23,24) | AC/MTX | 37 | 0 | 0 | 3 (8.1) | 34 (91.9) |
| NCI(25,26) | AC/MTX | 30† | NA | NA | NA | NA |
| NCI(25,26) | AC/MTX | 21‡ | NA | NA | NA | NA |
| EORTC(27) | CYVADIC | 145 | 19 (13.1) | 34 (23.5) | 16 (11.0) | 76 (52.4) |
| Bergonié(28) | CYVADIC | 31 | 0 | 1 (3.2) | 20 (64.5) | 10 (32.3) |
| All Dox + other | Dox + other | 314 | 19/243 (7.8) | 40/243 (16.5) | 41/243 (16.9) | 143/243 (58.8) |

Note: NA, data not available.
* Chemotherapy: please see footnote in Table 1.
† Includes 13 patients treated outside the randomized trial.
‡ Includes 13 patients treated outside the randomized trial.

by G-CSF. After a median follow-up of 59 months, an intention to treat analysis showed median disease-free survival times of 48 versus 16 months ($P$=0.04) and median overall survival times of 75 versus 46 months ($p$ = 0.03) for chemotherapy and control groups, respectively. It should be noted that although there is a delay in distant relapse in the chemotherapy arm, at 4 years the distant relapse rates are the same between the arms (44 and 45%). There remains a significant difference in 4-year overall survival with an absolute number of seven fewer deaths due to tumour in the chemotherapy arm. However, although 5-year median follow-up may be sufficient to observe the majority of distant relapses in high grade STS, improved treatments in supportive care may extend survival for patients with metastases, and it is possible they may die at different rates in the two arms; only longer follow-up will clarify this point. The toxicity was mainly hematological: 35% of patients had grade 4 leukopenia, 33% developed neutropenic sepsis, 24% required packed red blood cell transfusions and 4% had grade 4 thrombocytopenia. No cardiotoxicity was observed using ventricular ejection fraction measurements. Seven patients did not receive the prescribed chemotherapy and four patients did not complete treatment due to toxicity after two, three, four and five courses of treatment.

Another Italian group from Siena performed an adjuvant chemotherapy trial on 88 patients randomized after surgery to observation or chemotherapy.[31] The chemotherapy consisted of epirubicin during the first half of the study, and epirubicin combined with ifosfamide for the rest of the study. Overall, among the 81 evaluable patients, patients on adjuvant chemotherapy had improved 5-year disease-free survival (65 vs. 41%; $p$ = 0.01) and a trend for overall survival (72 vs. 47%; $p$ = 0.06) compared to patients not receiving chemotherapy. It should be noted that only 19 patients received an intensive epirubicin/ifosfamide combination; the remainder in the chemotherapy arm received single agent epirubicin. Given that this small study is reported only in abstract form, it should be interpreted with caution.

### *Practitioner feedback*

The draft recommendations were sent to 78 practitioners in Ontario. The sample consisted of 26 medical oncologists, 14 radiation oncologists, 32 surgeons, four gynecologists and two pathologists. Of the 46 (61%) surveys returned, 88% agreed with the recommendations and 70% approved the recommendations as a practice guideline. Six (18%) respondents provided written comments. These comments were reviewed by the members of the Sarcoma DSG, and modifications were made to the document to address these comments.

One practitioner commented that uterine sarcomas have traditionally been treated somewhat differently than other STS, rightly or wrongly. This practitioner felt that the effectiveness of the guideline could be improved by: (1) providing a rationale for including uterine STS in the current analysis and draft recommendations, or (2) excluding uterine sarcomas from the analysis, or (3) making specific comments in the document about these tumours. As a result of this comment, a statement that no specific recommendation can be made about uterine sarcomas was added to the Practice Guideline.

Another practitioner commented that assessing tumour grade and its role in tumour responsiveness should be considered. The Sarcoma DSG considered that the guideline document already addressed this issue.

### Discussion

The benefits in preventing disease relapse and improving patient survival, especially in patients with resected STS of the extremities, although modest, compare favourably with results for adjuvant chemotherapy in early breast cancer[32,33] and stage III colon cancer,[34] where adjuvant chemotherapy is considered standard care. The SMAC database used to draw these conclusions is, however, much smaller than similar databases for the other common tumours. With this limitation, doxorubicin-based adjuvant chemotherapy can be reasonably considered for adult patients with resected STS of the extremities at high risk for recurrence (deep high grade tumours > 5 cm in size)[1,5] and at low risk of adverse effects (no underlying diseases, particularly cardiovascular).

Despite some variation in the results of individual trials, all meta-analyses have shown that in patients with resected STS of any type, doxorubicin-based adjuvant chemotherapy significantly prolongs disease-free survival. The 10% absolute increase in disease-free survival at 10 years shown in the SMAC individual patient data overview is due both to reduced distant metastases and local recurrences. In spite of this reduction of relapses, there is only a trend for increased survival for the entire group of sarcoma patients (Table 2).

Although the data are consistent with modest survival benefits across all sarcomas examined, there are clues as to potential patient subgroups which are more likely to benefit. In the SMAC meta-analysis, a statistically significant survival benefit associated with doxorubicin-based chemotherapy (7% absolute increase in survival at 10 years) was found in 886 patients with extremity sarcomas. The next two largest groups are those of uterine and trunk sarcomas with 263 and 182 patients, respectively. Few retroperitoneal sarcomas are included, and the main contributing study[26] was terminated early because of an adverse effect of chemotherapy and substantial toxicity.

Only 30 patients in the SMAC meta-analysis had abdominal sarcomas.[14] Furthermore, only 24% of patients in the database were above age 60. Many of the trials excluded patients above age 65, or in some cases 70. Thus, it is difficult to generalize the beneficial results detected in the SMAC overview to patients with retroperitoneal tumours, or those above the age of 70 years. Finally, there was no difference in the reduction of mortality or disease recurrence between single-agent or combination adjuvant therapy with doxorubicin.

The information related to the effect of chemotherapy on local control should be interpreted with caution. In the SMAC meta-analysis, 15% of patients treated with chemotherapy experienced local recurrence (101 of 659 cases) compared with 19% (126 of 656 cases) in the control group. These relatively high rates of local recurrence are of concern. Local treatment is most important in the initial management of sarcomas. In extremity sarcomas, wide surgical excision, supplemented by radiation in cases where tumour size or location limits the procedure, can achieve local control in over 90% of cases.[35–38] However, the meta-analysis did not separately assess the local recurrence rate for extremity sarcomas. The higher rate of local recurrence reported may be explained by the inclusion of non-extremity sarcomas, which are known to have a higher rate of local failure.[35]

The consideration of doxorubicin-based adjuvant chemotherapy for patients with STS at non-extremity locations is more problematic. There are limited data about these tumours, and the available trials are inconclusive as to benefits with respect to disease relapse or survival. The data on uterine sarcomas consist mostly of patients investigated in a single trial with negative results; therefore, no specific recommendations can be made about these tumours. Few trials investigated patients with retroperitoneal sarcomas, and the observed adverse effect of chemotherapy when combined with radiation, would suggest that doxorubicin-based adjuvant chemotherapy should not be recommended for retroperitoneal sarcomas. There are insufficient data on patients with gastrointestinal stromal tumours (GIST) to recommend doxorubicin-based adjuvant chemotherapy. GIST represent a discrete group of tumours with a mutation of the proto-oncogene c-kit and overexpression of the associated tyrosine kinase. Although these tumours are generally resistant to chemotherapy, a specific inhibitor STI 571 or imatinib, induces remission in approximately half the cases with advanced disease.[39] The role of this drug as an adjuvant has not yet been investigated.

Benefits in preventing disease relapse and improving survival are achieved at the cost of a significant degree of toxicity, including a 5% rate of life-threatening drug-induced events and a 2% toxic fatality rate. Although most toxicity is acute (for example, nausea and vomiting, infections, cardiac arrhythmias), other events, such as cardiomyopathy, have long-term consequences. All these untoward effects have a major impact on treatment compliance (Table 4) and will affect patient quality of life, which unfortunately has not been specifically measured.

Strategies for reducing anthracycline-induced cardiotoxicity should be investigated.[40] This approach may have limitations, as the use of doxorubicin by infusion compared with the drug given by bolus reduced not only cardiotoxicity but also disease-free and overall survival.[41] Because of problems with toxicity and drug compliance, patient quality of life should be investigated and reported. As the meta-analyses have shown, a large number of patients will be required to demonstrate significant differences in outcomes, and these trials will require international cooperation.

In a meta-analysis of the 14 trials included in the SMAC report, the benefit for doxorubicin-based combination chemotherapy versus observation was similar to the benefit of single-agent doxorubicin versus observation. The combination regimens used in these trials are rarely used today. On the other hand, combination of doxorubicin with ifosfamide (the second most active drug in advanced STS) is only represented by a small study of 31 patients.[9] Three recently reported trials,[29–31] using dose-intensive ifosfamide with doxorubicin and dacarbazine[29] or epirubicin[30,31] under cover of G-CSF, have demonstrated either a trend[29,31] or a significant improvement[30] of disease-free and overall survival. This approach is also being investigated in a large EORTC-sponsored RCT, and it should be supported to determine precisely the potential advantage of combination chemotherapy.

### Practice guideline

### *Target population*

These recommendations apply to adult patients with completely resected soft tissue sarcoma.

### *Recommendations*

- It is reasonable to consider anthracycline-based adjuvant chemotherapy in patients who have had removal of a sarcoma with features predicting a high likelihood of relapse (deep location, size > 5 cm, high histological grade). These features correspond to International Union Against Cancer (UICC) stage III.
- Although the benefits of adjuvant chemotherapy are most apparent in patients with extremity sarcomas (7% RD for overall survival at 10 years), patients with high-risk tumours at other sites should also be considered for such therapy.

*Qualifying statements*

- There is insufficient evidence on patients with retroperitoneal sarcomas and stromal cell tumours of the gastrointestinal tract to make recommendations for adjuvant chemotherapy. The risk of serious toxicity when chemotherapy is combined with radiation therapy is of major concern. Data on uterine sarcomas derive mostly from a single trial with negative results; therefore, no specific recommendations can be made about these tumours.
- Risks of severe persistent adverse effects of adjuvant chemotherapy, such as cardiomyopathy, should be carefully evaluated and balanced against the expected benefit, particularly in patients aged 70 years or older and those with significant comorbidity.
- There are insufficient data to determine whether single-agent or combination doxorubicin chemotherapy should be recommended. This decision should take into account issues such as patient preference/convenience, likely adverse effects, costs and available resources.

*Key evidence*

- Considering all resected STS patients, doxorubicin-based adjuvant chemotherapy significantly reduces all recurrences, with an absolute benefit of 10% (95% CI, 5–15%; $p = 0.0001$) at 10 years. There is only a non-significant effect for survival, with an absolute benefit of 4% at 10 years (95% CI, -1 to 9%). Considering only patients with STS of the extremities, the benefit of adjuvant chemotherapy is 7% at 10 years ($p = 0.001$). A recently reported small randomized study using high-dose epirubicin and ifosfamide in large high-grade extremity sarcomas showed improved disease free ($p = 0.04$) and overall survival ($p = 0.03$). Most chemotherapy regimens produce significant toxicity.

*Future research*

- Patients should be encouraged to participate in clinical trials comparing adjuvant chemotherapy versus observation to further characterize benefits.

*Practice guideline date*

Completed 10 November 2000. Updated 13 June 2001.

Cancer Care Ontario Practice Guidelines Initiative (CCOPGI) practice guidelines are reviewed and updated regularly. Please visit the CCOPGI website at http://www.cancercare.on.ca/ccopgi/ for updates to this guideline.

### Acknowledgements

Sponsor: Cancer Care Ontario and the Ontario Ministry of Health and Long Term Care.

### References

1. Brennan ME, Alektiar KM, Maki RG. Soft tissue sarcoma. In: DeVita VT, Hellman S, Rosenberg SA, eds. *Cancer: Principles and Practice of Oncology*. 6th ed. Philadelphia–New York: JB Lippincott, 2001: 1841–90.
2. National Cancer Institute of Canada. *Canadian Cancer Statistics 2001*. Toronto, Canada; 2001: 76–7.
3. Lewis JJ, Benedetti F. Adjuvant therapy for soft tissue sarcoma. *Surg Oncol Clin N Am* 1997; 6: 847–62.
4. Mertens WC, Bramwell VHC. Adjuvant chemotherapy for soft tissue sarcomas. *Hematol Oncol Clin North Am* 1995; 9: 801–15.
5. Sobin LH, Wittekind Ch, eds. *International Union Against Cancer. TNM Classification of Malignant Tumours*. 5th ed. New York: Wiley-Liss, 1997: 106–9.
6. Jones GW, Chouinard E, Patel M. Adjuvant adriamycin (doxorubicin) in adult patients with soft-tissue sarcomas: a systematic overview and quantitative meta-analysis [Abstract]. *Clin Invest Med* 1991; 14: A772.
7. Zalupski MM, Ryan JR, Hussein ME, *et al.* Defining the role of adjuvant chemotherapy for patients with soft tissue sarcoma of the extremities. In: Salmon SE, ed. *Adjuvant Therapy of Cancer VII*. Philadelphia: JB Lippincott, 1993: 385–92.
8. Tierney JF, Mosseri V, Stewart LA, Souhami RL, Parmar MKB. Adjuvant chemotherapy for soft-tissue sarcoma: review and meta-analysis of the published results of randomized clinical trials. *Br J Cancer* 1995; 72: 469–75.
9. Sarcoma Meta-analysis Collaboration. Adjuvant chemotherapy for localized resectable soft-tissue sarcoma of adults: meta-analysis of individual data. *Lancet* 1997; 350: 1647–54.
10. Browman GP, Levine MN, Mohide EA, Hayward RSA, Pritchard KI, Gafni A, *et al.* The practice guidelines development cycle: A conceptual tool for practice guidelines development and implementation. *J Clin Oncol* 1995; 13: 502–12.
11. Omura GA, Blessing JA, Major F, Lifshitz S, Ehrlich CL, Mangan C, *et al.* A randomized trial of adjuvant adriamycin in uterine sarcomas: a Gynecologic Oncology Group study. *J Clin Oncol* 1985; 3: 1240–5.
12. Antman K, Suit H, Amato D, Corson J, Wood W, Proppe K, *et al.* Preliminary results of a randomized trial of adjuvant doxorubicin for sarcomas: lack of apparent difference between treatment groups. *J Clin Oncol* 1984; 2: 601–8.
13. Lerner HJ, Amato DA, Savlov E, DeWys WD, Mittelman A, Urtasun RC, *et al.* Eastern Cooperative Oncology Group: a comparison of adjuvant doxorubicin and observation for patients with localized soft tissue sarcoma. *J Clin Oncol* 1987; 5: 613–7.
14. Alvegard TA, Sigurdsson H, Mouridsen H, Solheim Ø, Unsgaard B, Ringborg U, *et al.* Adjuvant chemotherapy with doxorubicin in high grade soft tissue sarcoma: a randomized trial of the Scandinavian Sarcoma Group. *J Clin Oncol* 1989; 7: 1504–13.
15. Gherlinzoni F, Bacci G, Picci P, Capanna R, Calderoni P, Lorenzi EG, *et al.* A randomized trial for treatment of high grade soft tissue sarcoma of the extremities: preliminary results. *J Clin Oncol* 1986; 4: 552–8.

16. Picci P, Bacci G, Gherlinzoni F, Capanna R, Mercuri M, Rugieri P, *et al*. Results of a randomized trial for the treatment of localized soft tissue sarcoma (STS) of the extremities in adult patients. In: Ryan JR, Baker LH, eds. *Recent concepts in sarcoma treatment*. Dodrecht: Kluwer Academic Publishers, 1988: 144–8.
17. Antman K, Amato D, Pilepich M, Lerner H, Balcezak S, Borden E, *et al*. A preliminary analysis of a randomized Intergroup (SWOG, ECOG, CALGB, NCOG) trial of adjuvant doxorubicin for soft tissue sarcomas. In: Salmon SE, ed. *Adjuvant therapy of cancer*. Philadelphia: JB Lippincott, 1990: 725–34.
18. Baker LH. Adjuvant therapy for soft tissue sarcomas. In: Ryan JR, Baker LH, eds. *Recent concepts in sarcoma treatment*. Dodrecht: Kluwer Academic Press, 1988: 130–5.
19. Antman K, Ryan L, Borden E, *et al*. Pooled results from three randomized adjuvant studies of doxorubicin versus observation in soft tissue sarcoma: 10-year results and review of the literature. In: Salmon SE, ed. *Adjuvant Therapy of Cancer VI*. Philadelphia: JB Lippincott, 1990: 529–43.
20. Benjamin RS, Terjanian TO, Fenoglio CJ, Barkley HT, Evans HL, Murphy WK, *et al*. The importance of combination chemotherapy for adjuvant treatment of high-risk patients with soft tissue sarcomas of the extremities. In: Salmon SE, ed. *Adjuvant Therapy of Cancer V*. Orlando, FL: Grune and Stratton, 1987: 735–44.
21. Edmonson JH, Fleming TR, Ivins JC, Burgert O, Soule EH, O'Connell MJ, *et al*. Randomized study of systemic chemotherapy following complete excision of nonosseous sarcomas. *J Clin Oncol* 1984; 2: 1390–6.
22. Edmonson JH. Systemic chemotherapy following complete excision of nonosseous sarcomas: Mayo Clinic experience. *Cancer Treat Symp* 1985; 3: 89–97.
23. Rosenberg SA, Tepper J, Glatstein E, Costa J, Young R, Baker A, *et al*. Prospective randomized evaluation of adjuvant chemotherapy in adults with soft tissue sarcoma of the extremities. *Cancer* 1983; 52: 424–34.
24. Chang AE, Kinsella T, Glatstein E, Baker AR, Sindelar WF, Lotze MT, *et al*. Adjuvant chemotherapy for patients with high-grade soft tissue sarcomas of the extremities. *J Clin Oncol* 1988; 6: 1491–500.
25. Glenn J, Kinsella T, Glatstein E, Tepper J, Baker A, Sugarbaker P, *et al*. A randomized, prospective trial of adjuvant chemotherapy in adults with soft tissue sarcomas of the head and neck, breast and trunk. *Cancer* 1985; 55: 1206–14.
26. Glenn J, Sindelar W, Kinsella T, Glatstein E, Tepper J, Costa J, *et al*. Results of multimodality therapy of resectable soft tissue sarcomas of the retroperitoneum. *Surgery* 1985; 97: 316–25.
27. Bramwell V, Rouesse J, Steward W, Santoro A, Schraffordt-Koops H, Buesa J, *et al*. Adjuvant CYVADIC chemotherapy for adult soft tissue sarcoma: reduced local recurrence but no improvement in survival: a study of the European Organization for Research and Treatment of Cancer Soft Tissue and Bone Sarcoma Group. *J Clin Oncol* 1994; 12: 1137–49.
28. Ravaud A, Bui NB, Coindre JM, Kantor G, Stöckle E, Lagarde P, *et al*. Adjuvant chemotherapy with CYVADIC in high risk soft tissue sarcoma: a randomized prospective trial. In: Salmon SE, ed. *Adjuvant Therapy of Cancer VI*. Philadelphia: JB Lippincott, 1990: 556–66.
29. Brodowicz T, Schwameis E, Widder J, Amann G, Wiltschke C, Dominkus M, *et al*. for the Austrian Cooperative Soft Tissue Sarcoma Study Group. Intensified adjuvant IFADIC chemotherapy for adult soft tissue sarcoma: a prospective randomized feasibility trial. *Sarcoma* 2000; 4: 151–60.
30. Frustaci S, Gherlinzoni F, De Paoli A, Bonetti M, Azzarelli A, Comandone A, *et al*. Adjuvant chemotherapy for adult soft tissue sarcomas of the extremities and girdles: results of the Italian Randomized Cooperative Trial. *J Clin Oncol* 2001; 19: 1238–47.
31. Petrioli R, Coratti A, Correale P, D'Aniello C, Grimaldi L, Mancini S, *et al*. Epirubicin alone or epirubicin + ifosfamide as adjuvant chemotherapy in soft tissue sarcomas. *Ann Oncol* 2000; 11(2): 60 (abstract G16).
32. Anonymous. Clinical Practice Guidelines for the Care and Treatment of Breast Cancer. A Canadian consensus document. *Can Med Assoc J* 1998; 158 (Suppl 3): S43–63.
33. Early Breast Cancer Trialists' Collaborative Group. Systematic treatment of early breast cancer by hormonal, cytotoxic, or immune therapy: 133 randomized trials involving 31,000 recurrences and 24,000 deaths among 75,000 women. *Lancet* 1992; 339: 1–15, 71–85.
34. Figueredo A, Fine S, Maroun J, Walker-Dilks C, Wong S, and the Provincial Gastrointestinal Disease Site Group. Adjuvant therapy for stage III colon cancer after complete resection. *Cancer Prev Control* 1997; 1: 304–19.
35. LeVay J, O'Sullivan B, Catton CN, Bell RS, Fornasier VL, Cummings B, *et al*. Outcome and prognostic factors in soft tissue sarcoma in the adult. *Int J Radiat Oncol Biol Phys* 1993; 27: 1091–9.
36. Wilson AN, Davis A, Bell RS, O'Sullivan B, Catton C, Madadi F, *et al*. Local control of soft tissue sarcoma of the extremity: The experience of a multidisciplinary sarcoma group with definitive surgery and radiotherapy. *Eur J Cancer* 1994; 30A: 746–51.
37. Noria S, Davis AM, Kandel RA, Levesque J, O'Sullivan B, Catton CN, *et al*. Residual disease following unplanned excision of a soft tissue sarcoma of an extremity. *J Bone Joint Surg Am* 1996; 78A: 650–5.
38. Davis AM, Kandel RA, Wunder JS, Unger R, Meer J, O'Sullivan B, *et al*. Unplanned excision as a predictor of local relapse in soft tissue sarcoma of the extremity. *J Surg Oncol* 1997; 66: 81–7.
39. van Oosterom AT, Judson I, Verweij J, Stroobants S, di Paola ED, Dimitrijvic S, *et al*. Safety and efficacy of imatinib (STI571) in metastatic gastrointestinal stromal tumours: a phase I study. *Lancet* 2001; 358: 1421–3.
40. Allen A. The cardiotoxicity of chemotherapeutic drugs. *Semin Oncol* 1992; 19: 529–42.
41. Casper ES, Gaynor JJ, Hajdu SI, Magill GB, Tan C, Friedrich C, *et al*. A prospective randomized trial of adjuvant chemotherapy with bolus versus continuous infusion of doxorubicin in patients with high-grade extremity soft tissue sarcoma and an analysis of prognostic factors. *Cancer* 1991; 68: 1221–9.

**Appendix 1.** Staging System for Soft Tissue Sarcoma

**STAGE GROUPING**

| | | | | |
|---|---|---|---|---|
| Stage IA | G1,2 | T1a | N0 | M0 |
| | G1,2 | T1b | N0 | M0 |
| Stage IB | G1,2 | T2a | N0 | M0 |
| Stage IIA | G1,2 | T2b | N0 | M0 |
| Stage IIB | G3,4 | T1a | N0 | M0 |
| | G3,4 | T1b | N0 | M0 |
| Stage IIC | G3,4 | T2a | N0 | M0 |
| Stage III | G3,4 | T2b | N0 | M0 |
| Stage IV | Any G | Any T | N1 | M0 |
| | Any G | Any T | Any N | M1 |

**TNM CLINICAL CLASSIFICATION**

**T-Primary Tumour**

| | | |
|---|---|---|
| TX | | Primary tumour cannot be assessed |
| T0 | | No evidence of primary tumour |
| T1 | | Tumour 5 cm or less in greatest dimension |
| | T1a | Superficial tumour* |
| | T1b | Deep tumour* |
| T2 | | Tumour more than 5 cm in greatest dimension |
| | T2a | Superficial tumour* |
| | T2b | Deep tumour* |

Note: *Superficial tumour is located exclusively above the superficial fascia without invasion of the fascia; deep tumour is located either exclusively beneath the superficial fascia or superficial to the fascia with invasion of or through the fascia. Retroperitoneal, mediastinal, and pelvic sarcomas are classified as deep tumours.

**N-Regional Lymph Nodes**

| | |
|---|---|
| NX | Regional lymph nodes cannot be assessed |
| N0 | No regional lymph node metastasis |
| N1 | Regional lymph node metastasis |

**M-Distant Metastasis**

| | |
|---|---|
| MX | Distant metastasis cannot be assessed |
| M0 | No distant metastasis |
| M1 | Distant metastasis |

**G Histopathological Grading**

| | |
|---|---|
| GX | Grade of differentiation cannot be assessed |
| G1 | Well differentiated |
| G2 | Moderately differentiated |
| G3 | Poorly differentiated |
| G4 | Undifferentiated |

Source: International Union Against Cancer. TNM Classification of Malignant Tumours, fifth edition. New York: Wiley-Liss; 1997.

**Appendix 2.** Trial Names and Chemotherapy Regimens Used in Published Studies

GOG[11] = Gynecological Oncology Group
Regimen: doxorubicin 60 mg/m$^2$ i.v. every 3 weeks for eight doses.
No concurrent RT.

DFCI[12] = Dana Farber Cancer Institute
Regimen: doxorubicin 90 mg/m$^2$ i.v. every 3 weeks for five doses.
Concurrent RT 62.5–67.5 Gy over 6.5–7.0 weeks, with reduced volumes after 45 Gy.

ECOG[13] = Eastern Cooperative Oncology Group
Regimen: doxorubicin 70 mg/m$^2$ i.v. every 3 weeks for seven doses.
No concurrent RT.

SSG[14] = Scandinavian Sarcoma Group
Regimen: doxorubicin 60 mg/m$^2$ i.v. every 4 weeks for nine cycles.
Concurrent RT in patients with marginal resection, 51 Gy in 17 fractions over 24 days; for retroperitoneal tumours only 42 Gy.

Rizzoli[15,16] = Instituto Ortopedico Rizzoli
Regimen: doxorubicin 25–30 mg/m$^2$ i.v. days 1, 2 and 3, every 3 weeks for six cycles (or a total dose of 450 mg/m$^2$).
Concurrent RT in patients with marginal resection, 45 Gy over 3 weeks.

Intergroup[18,19] = Intergroup Sarcoma Study Group
Regimen: doxorubicin 35 mg/m$^2$ (escalated to 45 mg/m$^2$) on days 1 and 2, every 3 weeks up to a total doxorubicin dose of 450 mg/m$^2$.
Concurrent RT (dose not given) for patients with 'conservative resection'.

MDAH[20] = M. D. Anderson Hospital
Regimen VACAR: Vincristine 2 mg every week for 9 weeks, then every 3 weeks on day 1; doxorubicin 60 mg/m$^2$ on day 2 (up to a total dose 420 mg/m$^2$, and cyclophosphamide 200 mg/m$^2$ orally days 3–5; actinomycin D 0.3 mg/m$^2$ i.v. days 1–5 (maximal single dose 0.5 mg) after doxorubicin total dose achieved. Cycles every 4 weeks while on doxorubicin, then every 8 weeks; total duration of chemotherapy 2 years.
Concurrent RT 55 Gy over 6.5 weeks plus 10 Gy to scar.

Mayo[21,22] = Mayo Clinic
Regimen VAC/VAD: (1) VAC: vincristine 1.2 mg/m$^2$ days 1 and 5, cyclophosphamide 250 mg/m$^2$ on days 1, 3 and 5, and actinomycin D 0.325 mg/m$^2$ days 1–5; and (2) VAD: vincristine 1.2 mg/m$^2$ days 22 and 26, doxorubicin 50 mg/m$^2$ on day 24, and DTIC 250 mg/m$^2$ on days 22–26.
All drugs given i.v., in 6-week cycles, repeated six times.
No concurrent RT.
In addition, seven patients received MER-BCG on day 1 of each cycle of chemotherapy; then discontinued because chronic painful ulcers.

NCI[23–26] = National Cancer Institute, Bethesda, USA
Regimen AC/MTX: (1) AC: doxorubicin 50 mg/m$^2$ (escalated up to 70 mg/m$^2$) and cyclophosphamide 500 mg/m$^2$. Both i.v. day 1; cycles every 28 days until reaching a total doxorubicin dose of 550 mg/m$^2$.
(2) MTX: Afterwards, methotrexate 50 mg/m$^2$ (escalated up to 250 mg/m$^2$) i.v. infusion over 6 h on day 1, followed within 2 h by leucovorin 15 mg i.v. every 6 h for eight doses, or more if methotrexate blood level $>4\times10^{-7}$; cycles every 4 weeks up to a total maximal methotrexate dose of 1000 mg/kg.
Concurrent RT 60 Gy in 30–35 fractions; field size reduced after 45 Gy.
Six patients also received immunotherapy with *Corynebacterium parvum*.

EORTC[27] = European Organization for Research and Treatment of Cancer
Regimen CYVADIC: cyclophosphamide 500 mg/m$^2$, vincristine 1.5 mg/m$^2$ (maximum dose 2 mg) and doxorubicin 50 mg/m$^2$ on day 1, and DTIC 400 mg/m$^2$ on days 1–3. All drugs given i.v. every 4 weeks, eight times.
Concurrent RT in some patients, 40 Gy in 4 weeks for pelvic tumours, and 50 Gy for tumours outside the pelvis.

Bergonié[28] = Fondation Bergonié
Regimen CYVADIC: Drug doses as in EORTC regimen but cycles every 3 weeks, initially planned for 11 cycles, reduced to nine cycles after four patients had major toxicity.
Concurrent RT 50 Gy in 5 weeks plus 10–15 Gy boost to tumour bed. For retroperitoneal and deep abdominal tumours
RT dose decreased to 40 Gy over 5 weeks and 10 Gy boost; doxorubicin deleted during RT.

Brodowicz[29] = Austrian Cooperative Soft Tissue Sarcoma Study Group
Regimen IFADIC: ifosfamide 1500 mg/m$^2$ i.v. plus mesna on days 1–4, DTIC 200 mg/m$^2$ i.v. on days 1–4, and doxorubicin 25 mg/m$^2$ i.v. on days 1 and 2. IFADIC administered in a 14-day cycle for a total of six cycles. G-CSF ($30\times10^6$ IU/day) was injected s.c. on days 5–13. Concurrent RT during cycles 3 and 4, when doxorubicin is omitted. RT dose 50–60 Gy in twice daily fractions of 1.7 Gy.

Frustaci[30] = Italian Randomized Cooperative Trial
Regimen: epirubicin 60 mg/m$^2$ i.v. on days 1 and 2 and ifosfamide 1800 mg/m$^2$ mesna i.v. days 1–5, repeated every 3 weeks for five cycles; G-CSF rescue. RT given as either post-operative course (64–66 cGY in 33 or 34 fractions, or a pre-operative course (44.8 cGy in 28 fractions)

**Appendix 2.** Trial Names and Chemotherapy Regimens Used in Published Studies

Petrioli[31] = University of Siena

Regimen: epirubicin 75 mg/m$^2$ i.v. every 21 days; or epirubicin 20 mg/m$^2$ i.v. daily for 3 days and ifosfamide 1200 mg/m$^2$ i.v. daily for 5 days plus mesna; both regimens for four cycles. RT given to some patients: 55–60 cGy for extremity sarcomas, and 45–50 cGy for retroperitoneal sarcomas.
